# Mapping Genome Variants Sheds Light on Genetic and Phenotypic Differentiation in Chinese

**DOI:** 10.1016/j.gpb.2019.09.001

**Published:** 2019-09-09

**Authors:** Li Guo, Kai Ye

**Affiliations:** 1 MOE Key Laboratory for Intelligent Networks and Network Security, Faculty of Electronic and Information Engineering, Xi’an Jiaotong University, Xi’an 710049, China; 2 The School of Life Science and Technology, Xi’an Jiaotong University, Xi’an 710049, China; 3 The First Affiliated Hospital of Xi’an Jiaotong University, Xi’an 710061, China

Every human being looks different in one way or the other. That’s the work of genetic variations, the ultimate driving force for evolution as well as the cause for many human diseases. Mapping human genetic variants reveals global genetic diversity, and pinpoints causal variants behind genetic disorders. In recent years, several international consortiums, including the International HapMap Project [1], 1000 Genomes Project (1KGP) [2], [3], [4], [5], UK10K [6], Genome of the Netherlands (GoNL) [7], 1KJPN [8], and the Chinese Academy of Sciences Precision Medicine Initiative (CASPMI), reported in this issue [9], have all embarked on a journey to characterize the full spectrum of human genetic variants, This has been much powered by high-throughput sequencing technologies, such as next-generation sequencing (NGS), PacBio, and Oxford Nanopore, and bioinformatics algorithms for variant detections. To date millions of variants including single or multiple nucleotide substitutions, indels, and structural variants (SVs) have been detected in genomes of diverse ethnic origins, serving as valuable resources for genome-wide association studies (GWAS) to link genetic loci with diseases and providing guidance to personalized and precision treatments.

China has a population of about 1.4 billion, the world’s largest population with rather diverse ethnic groups. Han, the largest ethnic group in China and the world, constitutes about 18% of world population [10]. It is conceivable that characterizing genetic variations in various Chinese ethnic groups shall provide a vital foundation for elucidating the genetic basis of group-specific traits and disease susceptibility. Although variant discovery routinely involves mapping whole-genome or whole-exome sequencing data against the reference genome (GRCh38) of Caucasian genetic background produced by the Human Genome Project, this universal choice of reference genome is not suitable for capturing genetic variants in every ethnic group, calling for population-specific reference genomes or alternatively graph genomes [11]. Therefore, a high-quality Han-specific reference genome is badly needed for mapping Han genetic variants. Although two reference genomes of southern Han Chinese (HX1 and YH) are available, due to the genetic differences between northern and southern Han populations, a specific reference genome for northern Han is desired for categorizing genetic variations for the northerners, but so far nonexistent.

Reporting the nearly completed phase I of CASPMI launched in 2016, Du et al. [9] released a whole genome assembly NH1.0 for a northern Han individual combining multiple sequencing technologies including 10X Genomics, PacBio, and Bionano optical mapping. NH1.0 genome (Scaffold N50 of 46.63 Mb) is more continuous than the two existing Chinese genomes HX1 (21.98 Mb) and YH2.0 (20.52 Mb), also with higher integrity at chromosome level [9]. Comparing NH1.0 with GRCh38 reference genome identified 749 novel sequences spanning 4.76 Mb, harboring 2.6 million genetic variants including single-nucleotide variants (SNVs), small indels, and SVs. Understandably, NH1.0 is more representative to the Chinese population than GRCh38, revealed by a lower mismatch rate when mapping the WGS data of Han Chinese in the 1KGP to NH1.0 than to GRCh38. Overall, NH1.0 is a high-quality genome assembly that is invaluable to genetic studies of northern Han Chinese.

In addition to NH1.0 and the two existing southern Han genomes, Du et al. set out to perform variant discovery in Chinese population by mapping Illumina paired-end whole-genome sequencing (WGS) data (25–30 × coverage) of 597 Chinese participants. They detected a total of 28.8 million genome variations including SNVs and indels, nearly 11.75 million of which are novel variants found neither in dbSNP nor by the 1KGP. SVs are prevalent in human genomes as shown by many recent studies. Du et al. identified 106,382 indels in 597 participants, among which 65,847 are novel SVs not found in dbVar and DGV database. They also found 1432 copy number variations (CNVs) and most were low-frequency variants (<5%). In fact, most SVs they found were rare variants with allele frequencies lower than 0.5%. GWAS catalog mapping showed genes affected by these SVs are enriched in body mass index (BMI) and obesity-related pathways, suggesting their likely contribution to specific metabolism-related traits in Chinese. This large number of novel variants will support population and biomedical studies in China.

To understand the genetic variants associated with distinctive biological characteristics in Han Chinese populations, Du et al. compared variants in Chinese with outgroups (Americans, Africans, and Europeans, *etc*.) and discovered 55,271 SNVs and 6774 indels that are specific to Chinese. Metabolic pathway enrichment analysis and comparison with published GWAS catalog suggested that these population-specific variants are highly associated with metabolism-related traits and diseases, such as waist circumference, BMI, lipid metabolism, and diabetes. Particularly a SNV (rs1549293; T allele) at *KAT8* gene and another SNV (rs2398162) in the long non-coding RNA *NR2F2-AS1* are associated with male waist circumference and female hypertension, respectively. Northern Han males carrying the homozygous TT genotype have significantly larger waist measurements than their southern counterparts. Additionally, Du et al. conducted multi-omics data mining by combining DNase-seq, chromatin interaction analysis by paired-end tag sequencing (ChIA-PET), RNA-seq data in human Encyclopedia of DNA Elements (ENCODE), and the Genotype-Tissue Expression (GTEx) data. They provided mechanistic evidence that the phenotypic consequence conferred by TT alleles is likely attributed to alterations in epigenetics and gene expression of two obesity-associated genes *FUS* and *HSD3B7*, since rs1549293 resides in the enhancer regions of these genes.

Northern and southern Chinese are different in traits such as body build and waist circumference [9]. Du et al went further to identify variants that may contribute to both genetic and phenotypic differentiation between northern and southern Chinese. They used principle component analysis to analyze the genetic structure of Han population and showed that southern and northern Han belonged to distinct clusters, reflecting clear genetic differentiation between these two groups. Further fixation index (*F_st_*) analysis identified hotspots across four chromosomes. Interestingly, two peaks were observed in chromosome 6 around regions encoding the major histocompatibility complex, possibly correlated with exposure of the two populations to diverse climate and diet conditions. The *F_st_* analysis also illuminated the genetic basis of observed difference in body build between these two populations, by finding several strong variant differentiation signatures located in genes that are related to metabolism of fatty acids (*FADS1*, *FADS2*, and *FADS3*), cholesterol (*LILRA3*) and folate (*MTHFR* and *TCN2*) on various chromosomal locations. Finally, Du et al. analyzed the novel variations identified in CASPMI population for mutational signatures using non-negative matrix factorization, and found five signatures representative of different mutational processes in catalog of somatic mutations in cancer. The mutational spectrum was found overall similar between northerners and southerners, and the different load of mutational signatures were likely related to different average generation time in these two populations.

In short, Du et al. successfully produced a high-quality genome assembly of northern Han Chinese and comprehensively categorized genetic variants of 597 Chinese individuals from deep sequencing data. The variant and phenotype association analysis identified differential SNVs that might contribute to different traits of northern and southern Chinese. This study represents a significant progress of population genetic investigation of Chinese. Yet as demonstrated by several recent variant studies, a more comprehensive catalog of genetic variants in Chinese population is likely to be achieved in the future, when more participants, combination of high coverage long-read sequencing and NGS data, and improved variant calling framework are incorporated. When that day comes, fulfilling precision medicine for Chinese may not be far away.

## Competing interests

None declared.
